# Corneal irregularity and visual function using anterior segment optical coherence tomography in TGFBI corneal dystrophy

**DOI:** 10.1038/s41598-022-17738-3

**Published:** 2022-08-12

**Authors:** Yuito Abe, Takashi Omoto, Kohdai Kitamoto, Tetsuya Toyono, Junko Yoshida, Ryo Asaoka, Satoru Yamagami, Takashi Miyai, Tomohiko Usui

**Affiliations:** 1 Department of Ophthalmology, University of Tokyo Graduate School of Medicine, 7-3-1 Hongo, Bunkyo-ku, Tokyo, 113-8655 Japan; 2 Department of Ophthalmology, Kanto Central Hospital for Public School Teachers, 6-25-1 Kamiyoga, Setagaya-ku, Tokyo, 158-8531 Japan; 3 Department of Ophthalmology, International University of Health and Welfare, 852 Hatakeda, Narita-shi, Chiba, 286-0124 Japan; 4 Department of Ophthalmology, Seirei Hamamatsu General Hospital, 2-12-12 Sumiyoshi, Naka-ku, Hamamatsu-shi, Shizuoka, 430-8558 Japan; 5 Department of Ophthalmology, Nihon University School of Medicine, 30-1 Oyaguchikamicho, Itabashi-ku, Tokyo, 173-8610 Japan

**Keywords:** Corneal diseases, Hereditary eye disease, Refractive errors, Vision disorders, Eye diseases, Eye abnormalities

## Abstract

The purpose of this study was to evaluate corneal irregular astigmatism of patients with granular and lattice corneal dystrophy (GCD and LCD). 70 GCD, 35 LCD, and 81 control eyes were included. Anterior and posterior corneal topographic data obtained from anterior segment optical coherence tomography were expanded into four components via Fourier harmonic analysis. These components were compared with healthy eyes and the association between each component and best-corrected visual acuity (BCVA) was investigated. Anterior and posterior components increased in both GCD and LCD eyes. Anterior and posterior components of GCD2, anterior of LCD type 1 (LCD1), posterior of LCD type IIIA (LCD 3A), and type IV (LCD4) significantly increased. BCVA was significantly associated with anterior and posterior components in LCD eyes but not in GCD. The anterior components of LCD1, anterior and posterior of LCD3A, and posterior of LCD4 , were positively correlated with BCVA. As conclusions, in GCD eyes, anterior and posterior components differed from those of the control but BCVA was not significantly associated with them. In LCD eyes, the anterior and posterior components increased, and BCVA was significantly associated with the anterior and posterior components.

## Introduction

Transforming growth factor beta-induced (TGFBI) corneal dystrophy is a bilateral corneal disease characterized by an abnormal deposition of extracellular matrix^1–9^. Its clinical characteristics differ per disease, and lesions can appear in each corneal layer^3^. Patients with TGFBI dystrophy present with reduced best-corrected visual acuity (BCVA) with disease progression. However, the severity of opacity and visual function might not always be correlated^10,11^.

With recent technological advancements, images and accurate information about the cornea and anterior segment of the eyes can be obtained. Corneal irregularities are evaluated by analyzing wavefront aberrations, which can explain the decrease in BCVA and contrast sensitivity in normal and diseased eyes^10–18^. Recently , with the advent of anterior segment optical coherence tomography (AS-OCT) using infrared light with high depth of field, irregular astigmatisms in the anterior and posterior surfaces, even in cloudy corneas, can now be quantified. Fourier analysis is performed to quantify the shape analysis of the anterior segment, which can explain low BCVA and contrast sensitivity^19^.

Stromal opacity and a higher-order aberration (HOA) of the cornea can affect BCVA in corneal dystrophy^10,20^. The current study focused on granular corneal dystrophy (GCD) and lattice corneal dystrophy (LCD), which account for most cases of TGFBI dystrophy. GCD and LCD are similar in a way that both are related to allelic mutations of the TGFBI gene^21–23^, whereas they are known to show quite different findings in the cornea; different types of deposits are observed in various layers^3^. However, only a few studies have investigated the anterior and posterior surfaces of the cornea in TGFBI dystrophy and its different subtypes in detail^10^. Therefore, in the current study, we aimed to evaluate anterior and posterior irregular astigmatism in GCD and LCD via a Fourier harmonic analysis of AS-OCT data.

## Results

**Figure 1:**
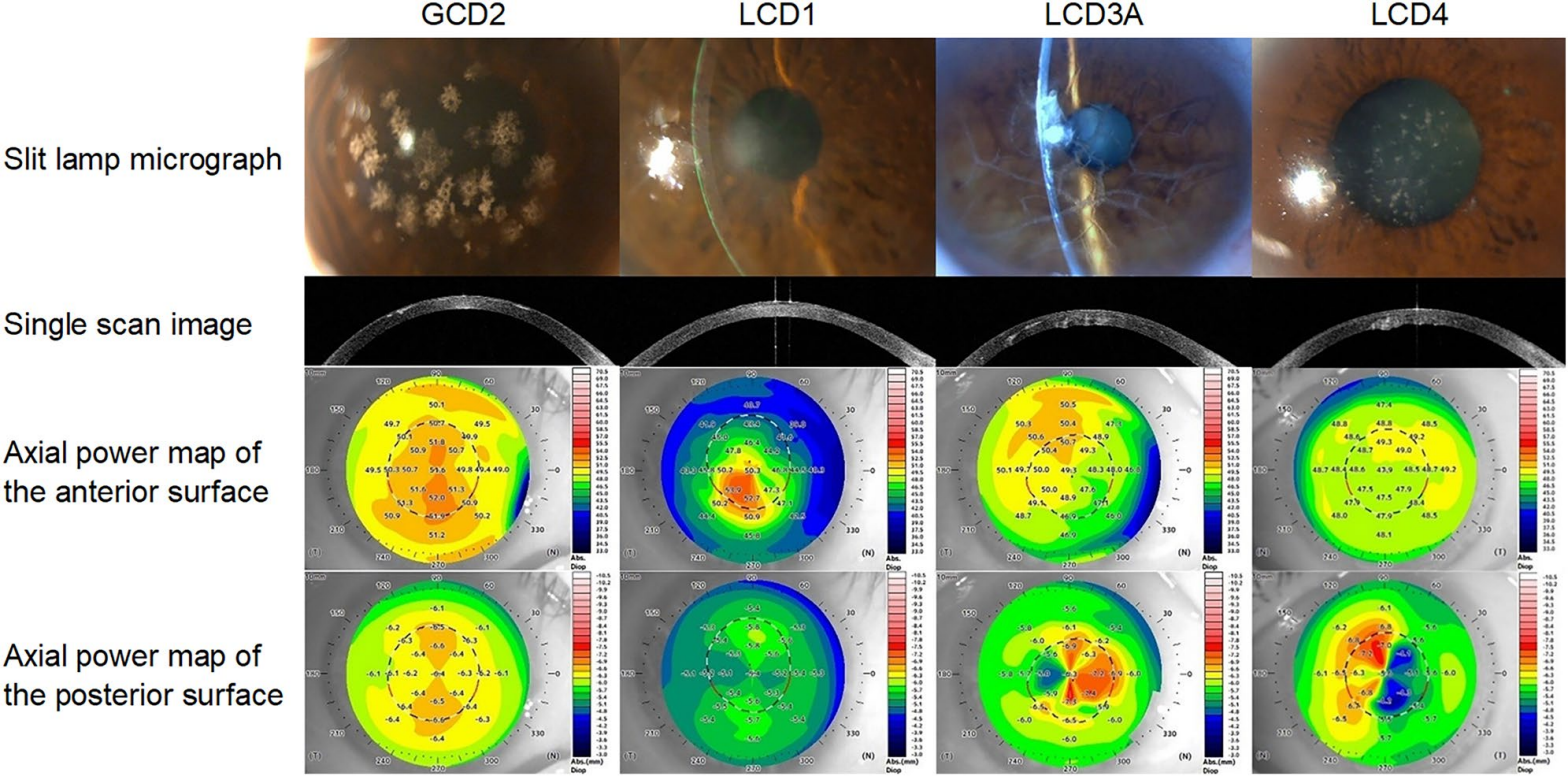
Representative example of AS-OCT image and topographic map in one case analyzed in this study. The BCVA of each case was 20/28, 20/400, 20/50 and 20/100. AS-OCT = anterior segment optical coherence tomography, BCVA = best-corrected visual acuity, GCD2 = granular corneal dystrophy type 2, LCD1 = lattice corneal dystrophy type 1, LCD3A = lattice corneal dystrophy type IIIA, LCD4 = lattice corneal dystrophy type IV.

In total, 70 GCD eyes in 37, 35 LCD eyes in 21, and 81 control eyes in 58 participants were included in the current study. Almost all of the GCD eyes were GCD type 2 (GCD2) (66 out of 70 eyes). GCD type 1 (GCD1) was excluded from the analysis owing to the statistical issues caused by the the lower number of samples (4 eyes). Thereafter, all GCD eyes were GCD2. The GCD and LCD groups had significantly worse BCVA than the control group. Table 1 and Fig. 1 show the characteristics of the participants and representative photos of the diseased groups, respectively. The values, ranges and P-values for each component is shown in Table 2.

**Table 1:** Demographic characteristics of the participants.

	Controls	GCD	LCD			
		GCD2	LCD (Total)	LCD1	LCD3A	LCD4
No. of eyes (patients)	81 (58)	66 (35)	35 (21)	11 (7)	11 (6)	13 (8)
Age (years)	66.3 ± 9.5	60.4 ± 16.8*	66.9 ± 14.9	51.1 ± 13.7*	75.8 ± 7.8*	72.6 ± 9.9*
Sex (male/female)	41/40	26/40	24/11	6/5	9/2*	9/4
IOL eyes	0	14	10	2	5	3
BCVA (LogMAR)	− 0.061 ± 0.034	0.17 ± 0.23*	0.52 ± 0.51*	0.69 ± 0.58*	0.30 ± 0.20*	0.56 ± 0.56*

*GCD*: Granular corneal dystrophy, *GCD1*: Granular corneal dystrophy type 1, *GCD2*: Granular corneal dystrophy type 2, *LCD*: Lattice corneal dystrophy, *LCD1*: Lattice corneal dystrophy type 1, LCD3A: Lattice corneal dystrophy type IIIA, LCD4: Lattice corneal dystrophy type IV, *IOL*: Intraocular lens, *BCVA*: Best-corrected visual acuity. Values were shown in mean ± standard deviation manner.

*p* values were calculated using t-test or Fisher’s exact test, appropriately. *, shows the statically significant difference from the control.

**Table 2:** Each Fourier component.

		Anterior				Posterior			
		Spherical	Regular	Asymmetry	Higher-order irregularity	Spherical	Regular	Asymmetry	Higher-order irregularity
Normal	Mean ± SD (D)	49.0 ± 1.92	0.51 ± 0.32	0.32 ± 0.29	0.17 ± 0.10	− 6.21 ± 0.25	0.17 ± 0.07	0.05 ± 0.03	0.02 ± 0.01
	Range (D)	44.1, 53.9	0.10, 1.58	0.06, 1.92	0.06, 0.66	− 6.89, − 5.76	0.01, 0.35	0.00, 0.12	0.01, 0.05
GCD2	Mean + SD (D)	49.4 ± 2.12	0.81 ± 0.51	0.52 ± 0.49	0.21 ± 0.09	− 6.18 ± 0.30	0.17 ± 0.09	0.15 ± 0.15	0.06 ± 0.05
	Range (D)	44.5, 53.9	0.14, 2.81	0.09, 3.48	0.11, 0.68	− 6.77, − 5.50	0.04, 0.49	0.02, 0.78	0.02, 0.34
	*P* value	0.27	0.0015*	0.0023*	0.037*	0.67	0.90	< 0.001*	< 0.001*
LCD (total)	Mean ± SD (D)	48.0 ± 1.97	0.93 ± 0.84	0.91 ± 1.2	0.30 ± 0.21	− 5.93 ± 0.35	0.32 ± 0.27	0.57 ± 0.59	0.31 ± 0.43
	Range (D)	42.9, 51.7	0.06, 3.44	0.03, 5.17	0.11, 0.88	− 6.79, − 5.33	0.05, 1.13	0.04, 2.41	0.02, 1.85
	*P* value	0.056	< 0.001*	< 0.001*	< 0.001*	< 0.001*	< 0.001*	< 0.001*	< 0.001*
LCD1	Mean ± SD (D)	47.9 ± 2.08	1.72 ± 1.04	1.65 ± 1.76	0.46 ± 0.28	− 5.89 ± 0.31	0.24 ± 0.16	0.26 ± 0.47	0.06 ± 0.06
	Range (D)	44.3, 50.8	0.47, 3.44	0.14, 5.17	0.18, 0.88	− 6.23, − 5.36	0.07, 0.67	0.04, 1.65	0.02, 0.24
	*P* value	0.56	< 0.001*	< 0.001*	< 0.001*	0.068	0.66	0.14	0.98
LCD3	Mean ± SD (D)	47.8 ± 2.34	0.56 ± 0.36	0.44 ± 0.33	0.17 ± 0.05	− 5.98 ± 0.39	0.23 ± 0.18	0.45 ± 0.42	0.23 ± 0.25
	Range (D)	42.9, 50,5	0.06, 1.12	0.08, 1.24	0.12, 0.29	− 6.49, -5.33	0.05, 0.63	0.07, 1.45	0.02, 0.90
	*P* value	0.22	0.94	0.93	0.98	0.084	0.35	0.0012*	0.0070*
LCD4	Mean ± SD (D)	48.3 ± 1.63	0.58 ± 0.41	0.67 ± 0.81	0.26 ± 0.11	− 5.91 ± 0.37	0.47 ± 0.35	0.95 ± 0.62	0.58 ± 0.57
	Range (D)	46.6, 51.7	0.17, 1.33	0.03, 3.24	0.11, 0.45	− 6.79, − 5.45	0.06, 1.13	0.14, 2.41	0.08, 1.85
	*P* value	0.54	0.90	0.27	0.050	0.011*	< 0.001*	< 0.001*	< 0.001*

*SD*: Standard deviation, *GCD2*: Granular corneal dystrophy type 2, *LCD*: Lattice corneal dystrophy, *LCD1*: Lattice corneal dystrophy type 1, *LCD3*: Lattice corneal dystrophy type IIIA, *LCD4*: Lattice corneal dystrophy type IV.

*p* values were calculated using the linear mixed effect model. *, *p* value < 0.05.

**Figure 2:**
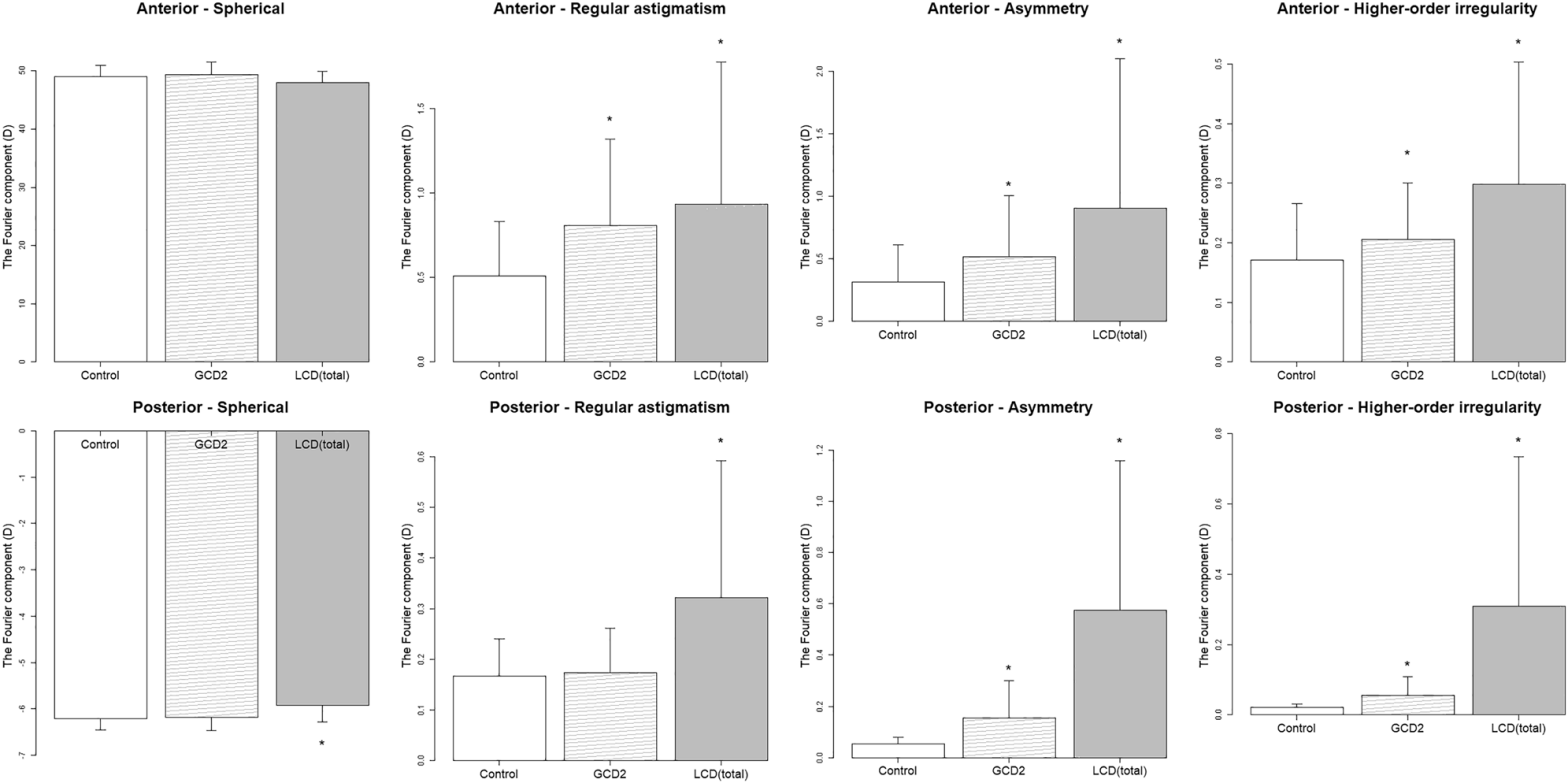
Fourier components for comparison between the phenotypes and controls. * indicates statistically significant difference. GCD = granular corneal dystrophy, LCD = lattice corneal dystrophy.

In the analysis according to phenotypes, when comparing the GCD and control eyes, the anterior (regular astigmatism, asymmetry, higher-order irregularity; *P* = 0.0015, = 0.0023 and = 0.036) and posterior (asymmetry, higher-order irregularity; *P* < 0.001 and *P* < 0.001) components significantly differed (Fig. 2). However, these components were not associated with BCVA (Table 3). Association between total corneal power and BCVA were also analyzed, but no significant differences emerged, similar to the anterior and posterior (Table 3).

**Table 3:** Association between BCVA and Fourier components.

		Anterior				Posterior				Total corneal			
		Spherical	Regular	Asymmetry	Higher-order irregularity	Spherical	Regular	Asymmetry	Higher-order irregularity	Spherical	Regular	Asymmetry	Higher-order irregularity
GC	Coefficient	− 0.016	0.020	0.061	− 0.11	0.039	− 0.16	0.017	− 0.085	− 0.020	0.025	0.056	− 0.21
D2	*P* value	0.20	0.67	0.40	0.72	0.66	0.58	0.90	0.84	0.17	0.63	0.47	0.41
LCD	Coefficient	− 0.054	0.28	0.27	1.55	0.19	0.40	− 0.026	0.39	− 0.050	0.37	0.22	0.57
(total)	*P* value	0.25	0.012*	< 0.001*	< 0.001*	0.39	0.13	0.87	0.029*	0.32	< 0.001*	< 0.001*	0.0011*
LCD1	Coefficient	− 0.060	0.33	0.31	1.50	0.44	− 1.42	− 0.30	− 3.28	− 0.06	0.37	0.31	1.54
	*P* value	0.50	0.046*	< 0.001*	0.064	0.60	0.33	0.52	0.35	0.52	0.02*	< 0.001*	0.061
LCD3	Coefficient	− 0.042	0.35	0.47	3.30	− 0.030	− 2.32	0.46	0.70	− 0.05	0.42	0.61	0.78
	*P* value	0.69	0.30	0.047*	0.036*	0.95	0.0048*	0.24	0.12	0.66	0.26	0.0020*	0.053
LCD4	Coefficient	− 0.10	0.15	0.18	1.59	0.23	0.67	0.11	0.43	− 0.07	0.56	0.12	0.48
	*P* value	0.40	0.65	0.18	0.12	0.49	0.010*	0.64	0.023*	0.53	0.11	0.32	0.016*

*BCVA*: Best-corrected visual acuity, *GCD2*: Granular corneal dystrophy type 2, *LCD (total)*: Lattice corneal dystrophy (Includes all subtypes), *LCD1*: Lattice corneal dystrophy type 1, *LCD3*: Lattice corneal dystrophy type III A, *LCD4*: Lattice corneal dystrophy type IV.

Coefficients and *p* values were calculated using the linear mixed effect model. *, *p* value < 0.05.

**Figure 3:**
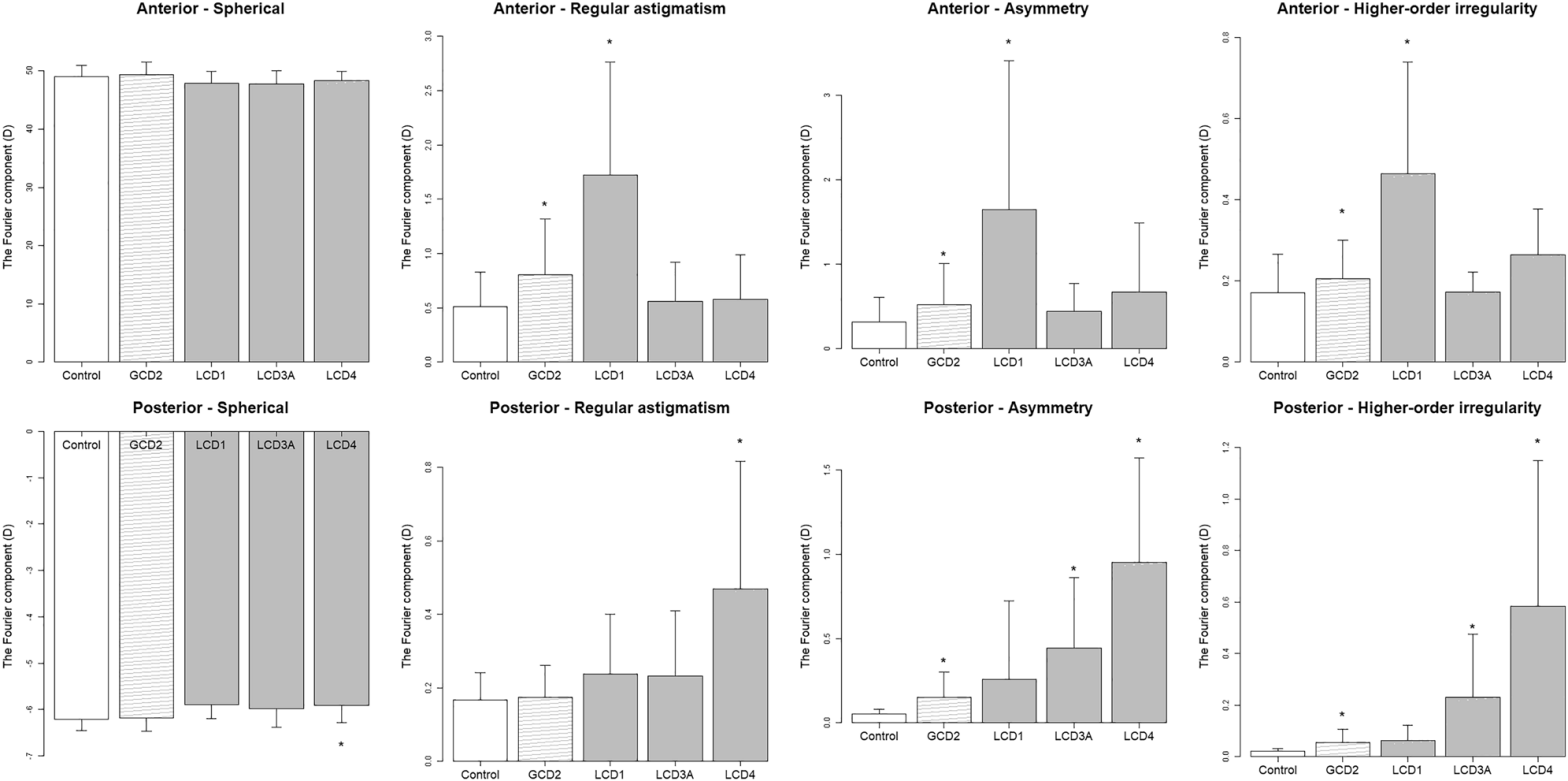
Fourier components for comparison between the subtypes and controls. * indicates statistically significant difference. GCD2 = granular corneal dystrophy type 2, LCD1 = lattice corneal dystrophy type 1, LCD3A = lattice corneal dystrophy type IIIA, LCD4 = lattice corneal dystrophy type IV.

In the comparison between the LCD and normal groups, the anterior (regular astigmatism, asymmetry, higher-order irregularity; *P* < 0.001, < 0.001 and < 0.001) and all of the posterior components (*P* < 0.001) significantly differed (Fig. 2). The Fourier components of the LCD eyes were significantly associated with BCVA in the anterior (regular astigmatism, asymmetry, and higher-order irregularity; *P* = 0.012, < 0.001, and < 0.001) and posterior (higher-order irregularity; *P* = 0.029) components (Table 3). In association between total corneal power and BCVA, positive associations were observed in regular astigmatism, asymmetry, and higher-order irregularity (*P* < 0.001, *P* < 0.001, and *P* = 0.0011) (Table 3). In the analysis according to LCD subtypes, the eyes with LCD type 1 (LCD1) had higher anterior components (regular astigmatism, asymmetry, and higher-order irregularity; *P* =  < 0.001, < 0.001 and < 0.001) (Fig. 3). Moreover, the anterior components (regular astigmatism and asymmetry; *P* = 0.046 and < 0.001) were positively associated with BCVA (Table 3). In association between total corneal power and BCVA, positive associations were similarly observed in regular astigmatism and asymmetry (*P* = 0.02 and < 0.001) (Table 3).

Only the posterior components of the eyes in patients with LCD type IIIA (LCD3A) significantly differed (asymmetry and higher-order irregularity; *P* = 0.0011 and = 0.0070) (Fig. 3). The anterior components (asymmetry and higher-order irregularity; *P* = 0.047 and = 0.036) were positively associated with BCVA. Meanwhile, there was a negative association between the posterior component (regular astigmatism; *P* = 0.0048) and BCVA (Table 3). In association between total corneal power and BCVA, positive associations were observed in asymmetry (*P* = 0.0020) (Table 3).

In LCD type IV (LCD4), the anterior components did not significantly differ, and all posterior components increased (*P* = 0.011, < 0.001, < 0.001 and < 0.001) (Fig. 3). In addition, there was a positive association only between the posterior components (regular astigmatism and higher-order irregularity; *P* = 0.010 and 0.023) and BCVA (Table 3). In association between total corneal power and BCVA, positive associations were observed in higher-order irregularity (*P* = 0.016) (Table 3).

The analysis results of the association between the central corneal thickness (CCT) and each component are shown in Table 4. In the anterior surface, significant association was only observed in the spherical component of LCD4, but there were no obvious associations in other components. In the posterior surface, positive associations were observed for total LCD and associations were observed in LCD3A and LCD4.

**Table 4:** Association between central corneal thickness and Fourier components.

		Anterior				Posterior			
		Spherical	Regular	Asymmetry	Higher-order irregularity	Spherical	Regular	Asymmetry	Higher-order irregularity
GCD2	Coefficient	3.45	− 1.16	0.90	27.3	28.8	− 53.9	− 9.53	− 35.3
	*P* value	0.12	0.78	0.87	0.23	0.034*	0.068	0.34	0.27
LCD (total)	Coefficient	6.44	0.25	5.50	22.3	− 35.4	88.4	43.9	103
	*P* value	0.29	0.99	0.58	0.73	0.23	0.014*	0.03*	< 0.001*
LCD1	Coefficient	− 4.53	5.39	8.54	28.8	12.2	− 15.6	11.7	− 123
	*P* value	0.35	0.58	0.25	0.52	0.78	0.85	0.65	0.54
LCD3	Coefficient	− 4.4	− 24.6	0.85	110	130	− 51.1	21.5	93.7
	*P* value	0.65	0.46	0.98	0.56	0.020*	0.48	0.42	0.032*
LCD4	Coefficient	40.3	37.3	9.56	68.5	− 72.1	109	85.6	106
	*P* value	0.012*	0.60	0.74	0.78	0.26	0.11	0.14	0.0037*

*GCD2*:  granular corneal dystrophy type 2, LCD (total):  Lattice corneal dystrophy (Includes all subtypes), *LCD1*: Lattice corneal dystrophy type 1, *LCD3*:  Lattice corneal dystrophy type IIIA, LCD4: Lattice corneal dystrophy type IV. Coefficients and *p* values were calculated using the linear mixed effect model. *, *p* value < 0.05.

When analyzed within 6-mm, GCD eyes showed similar results to those within 3-mm. LCD as a phenotype showed a significant increase in anterior spherical component (*P* = 0.026) (Supplemental Fig. 1, Supplemental Table 1), in addition to the significant difference observed within 3 mm. LCD1 eyes showed associations with BCVA in higher-order irregularity of anterior and total corneal power (*P* = 0.017, = 0.032) (Supplemental Table 2). There was a significant increase in the posterior spherical component in LCD3A (*P* = 0.046) (Supplemental Fig. 2, Supplemental Table 1), but there was relatively less association with BCVA (Supplemental Table 2). The LCD4 eyes also showed almost the same results.

## Discussion

The specific profile of the layer-by-layer Fourier components in GCD and LCD eyes, in addition to the association between them and BCVA, was discussed in this research. Moreover, in LCD, not only each phenotype but also each subtype was analyzed. GCD and LCD are known to show quite different findings in the cornea—both amyloid and hyaline deposits in GCD eyes—whereas amyloid deposits in LCD eyes and the depth of the deposits vary by the subtypes^3^. These findings were analyzed using AS-OCT in detail and these results should be beneficial for clinicians when evaluating visual function of the patients.

In GCD eyes, interstitial opacities between granular deposits were often confined to the anterior subepithelial layer (Fig. 1). Structural disturbances in the anterior and posterior surfaces were apparently smooth. However, there was a slight but significant increase in posterior irregular astigmatism (asymmetry, higher-order irregularity) in GCD2 eyes (Fig. 3). Interestingly, the posterior as well as the anterior components increased despite the relatively superficial existence of opacity. In a previous report by Yagi-Yaguchi et al., visual acuity was positively correlated with opacity grade, age, astigmatism in GCD2 eyes, but not with HOAs^10^. The HOA value in the correlation analysis in a previous study was not calculated layer-by-layer^10^. The current study analyzed the anterior and posterior components separately. However, the results were similar. That is, the Fourier components of the GCD eyes were not correlated with BCVA. Nevertheless, the GCD eyes had significantly lower BCVA than the control eyes. The factors causing a decrease in BCVA are challenging to analyze. However, the relatively low contributions of astigmatism factors reinforce the possibility that scattering caused by opacity may have a stronger effect on visual acuities.

In LCD eyes, there was an increase in Fourier components, and BCVA in the anterior and posterior surfaces was positively associated with these components. In the analysis according to each LCD subtype, the results differed between the subtypes and were characteristic to each subtype. As shown in Fig. 1, the cornea of one patient with LCD1 had stromal opacities with various reflectivity and sizes near the anterior surface.

The present analysis revealed that eyes with LCD1 showed an increased component in all anterior surfaces except spherical, which was also significant in the anterior surface in association with BCVA. These results were consistent with the previously reported pathological and optical microscopic findings^3,24^ In LCD1, phototherapeutics keratectomy (PTK) is sometimes performed in LCD1 because of a significant anterior surface lesion^25^. The best LCD1 treatment is surgical procedures, such as PTK and deep anterior lamellar keratoplasty, which are relatively limited to the anterior surface, considering only the astigmatism perspective.

A significant increase was found in the posterior surface components in LCD3A eyes. However, this was also found in both anterior and posterior surfaces in association with BCVA, and the results were not consistent and convincing. This subtype is previously reported to show an opacity from the stroma to the posterior surface in histopathological and light microscopic observations^3,26^. The increased posterior components in the present study appear compatible with these reports.

All posterior surface components increased in LCD4 eyes, and almost all were positively associated with BCVA. As shown in Fig. 1, the cornea of patients with LCD4 commonly presented with opacities in the posterior surface, which could be described as protrusions in the anterior chamber. Previous reports revealed that this subtype is characterized by deeper deposits without epithelial erosion^3,27,28^. The results of the current research were consistent with those of previous studies. In LCD4, penetrating keratoplasty has remained the primary treatment since it can improve BCVA because the posterior irregularity cannot be corrected using rigid gas permeable contact lenses, and the posterior opacity cannot be managed with PTK.

Each subtype of LCD had different characteristics. In each subtype, the results of the corneal shape analysis were similar to the typical AS-OCT imaging findings and were consistent with what has been noted clinically.

Previously, Yagi-Yaguchi et al. showed that visual acuity was correlated with HOAs, but not with grade, age, and astigmatism, in patients with LCD1, unlike GCD^10^. Similar to this report, we were able to show that higher-order irregularity is associated with BCVA. Additionally, evaluating the anterior and posterior subtypes independently was possible in the present study to indicate changes specific to each subtype. The factor of opacity scatter was not determined, and these astigmatism results alone do not determine the clinical treatment strategy. However, the presence of these astigmatisms and their associations with BCVA should be a factor that cannot be sufficiently ignored to contribute to treatment decisions, as mentioned above.

The current study had several limitations. Because of the nature of retrospective design, the differences of demographic characteristics among groups may have affected the results. The present study did only one measurement per examination. However, previous studies have reported the sufficiently high repeatability of CASIA measurements^29,30^. Corneal opacity is another important factor that affects patients’ BCVA in LCD and GCD. However, the degree of opacity was not evaluated in this study. As mentioned above, the effect of scattering caused by opacity on visual function cannot be ignored. Thus, the quantified data about opacity and its association with BCVA must be investigated. However, even considering this, a significant association was found between astigmatism and the reduction of BCVA in the present study, particularly in LCD eyes. However, no significant increase was found in astigmatism, which suggests the possibility of decreased BCVA cause by opacity, although GCD had a decreased BCVA. Some cases (23 cases) were not genetically diagnosed. Furthermore, some patients who underwent cataract surgery were included. The effect of cataract surgery on the postoperative corneal structure cannot be completely ruled out^31–33^. However, astigmatism caused by surgery is sufficiently weakened > 3 months postoperatively, according to previous reports^34–38^. Therefore, measurements performed at least three months postoperatively were adopted for eyes that had undergone cataract surgery. No eyes were wearing rigid contact lenses. In keratometry, tear film–stabilizing eye drops prior to keratometry measurements influenced K-readings significantly, especially in dry eyes^39^. In this study, the absence of criteria for the use of eye drops was a limitation. However, we think that the AS-OCT technique is unlikely affected by the tear fluid layer, including dry eye, as previously reported^40^.

In conclusion, in GCD eyes, subtype analysis showed a slight increase in the anterior and posterior components, but none of the components differed from the control, and BCVA was not significantly associated with these components. In LCD eyes, the anterior and posterior components increased, and BCVA was significantly associated with the anterior and posterior components. In the analysis according to each LCD subtype, the results differed between the subtypes in the following manner: only anterior components differed and anterior components positively associated with BCVA in LCD1; only the posterior components differed and the associations with BCVA were positive in anterior and negative in posterior components in LCD3A; posterior components increased and there was a positive association between posterior components and BCVA in LCD4.

## Methods

This cross-sectional study was approved by the clinical research review board of the University of Tokyo Hospital (20200006NI). Informed consent was obtained in the form of opt-out on the website for both participation in the study and publication of identifying information/images in an online open access publication, following the above-mentioned approval. This research was performed according to the tenets of the Declaration of Helsinki.

### Participants

Patient data were obtained from the University of Tokyo database. GCD and LCD patients who underwent AS-OCT imaging, in addition to routine examinations, such as slit-lamp microscopy, fundus examinations, and measurements of BCVA, were included. Patients with inaccurate diagnosis, corneal epithelial defects, corneal scarring, and a history of corneal surgery, such as PTK and corneal transplantation, which can affect the corneal surface, were excluded.

Of the 58 patients in total, 35 had genetic tests and were genetically diagnosed and the rest were not genetically examined but were clinically diagnosed by two corneal specialists (Y.A. and T.M.) and identified as dystrophy phenotypes and subtypes according to the International Classification of Corneal Dystrophies (IC3D) using the clinical information in the medical records^2,3^. Then, 66 eyes of 35 patients with GCD2, 11 eyes of 7 patients with LCD1, 11 eyes of 6 patients with LCD3A, and 13 eyes of 8 patients with LCD4 were included in the final analysis. Patients with LCD2 were excluded because the condition was different from TGFBI dystrophy^3^. Patients with other vision-affecting diseases were excluded from the analysis of the association between BCVA and Fourier components, but remained in other analyses (n = 9 eyes with GCD [cataract, glaucoma, and diabetic retinopathy] and n = 6 eyes with LCD [cataract, age-related macular degeneration, and central nervous system disorder]). GCD1 was excluded from the analysis because of the lower number of samples (4 eyes). Meanwhile, patients who had no corneal disease were included as normal controls. The criteria for normal eyes were no keratoconjunctival disease in both eyes, no hereditary disease, no previous laser treatment, astigmatism within − 1.5 diopter in keratometry, central corneal thickness between 500 and 560 μm, no rigid contact lenses use within 1 month, and BCVA of 1.0 or higher. In total, 81 eyes of 58 control patients were also included.

### OCT assessment

All AS-OCT images were obtained using SS-1000 CASIA or SS-2000 CASIA2 (Tomey Corporation, Inc., Aichi, Japan). The first measurements taken at the first hospital visit were selected. AS-OCT imaging provides more reliable and reproducible anterior segment assessment than other techniques, such as Scheimpflug technology. A previous report have also provided more reliable and reproducible anterior segment evaluation in keratometry and elevation data for the anterior and posterior corneal surfaces^41^. Additionally, CASIA2 is equipped with a quality statement (QS), an indicator that assesses whether the image has been correctly captured. The QS OK is only given if the anterior and posterior tracing lines are well defined. We only use the QS OK for the cornea in all scans. Furthermore, all images were manually checked for appropriate tracings. We used the “Corneal Map” mode, in which measurements were exported as data maps in cylindrical coordinates with a 22.5° angular separation. Corneal shape analysis was performed using two different layered assessments: axial power map of the anterior and posterior surfaces. Values within 3-mm and 6-mm diameter were analyzed. The data within 3-mm was used for primary analysis, because it is closer to the usual pupil diameter and assumed to be more important in association with visual function. Via a Fourier harmonic analysis, corneal dioptric data were expanded into four components as follows: spherical, regular astigmatism, asymmetry, and higher-order irregularity, according to the following formula:

$$F_{i} \left( \sigma \right) = a_{0} + c_{1} cos\left( {\sigma - \alpha_{1} } \right) + c_{2} cos2\left( {\sigma - \alpha_{2} } \right) + c_{3} cos3\left( {\sigma - \alpha_{3} } \right) + \cdots + c_{n} cosn\left( {\sigma - \alpha_{n} } \right)$$

in which a_0_ is the spherical component of the ring, 2 × c_1_ is the asymmetry, 2 × c_2_ is regular astigmatism, and the summation of c_3_ … c_n_ includes the higher-order irregularity.

### Statistical analysis

First, we compared the Fourier components between the phenotypes and control eyes. Second, the association between BCVA and the components was investigated. The association between CCT and the components was investigated, as well. Next, similar analyses were performed according to each subtype per phenotype.

TGFBI dystrophy, as is well known, develops bilaterally^1–3^. Therefore, we used the multivariate linear mixed-effects model where the random effect was the subjects, which were adjusted for age. The model adjusts for the hierarchical structure of the data, modeling in a way in which measurements are grouped within subjects to reduce the possible bias of including both eyes of one patient^42,43^. This was followed by Dunnett’s test for multiple comparisons when comparing the values between each sub-group in the phenotypes and the control^44^. A p value of 0.05 was considered statistically significant. All analyses were performed using R 4.0.2. (R Foundation for Statistical Computing, Vienna, Austria).

## Supplementary Information

Supplementary Information 1.

Supplementary Information 2.

Supplementary Information 3.

Supplementary Information 4.

## Data Availability

All data generated or analyzed during this study are included in this published article.
