# Supplementary material for: Corneal irregularity and visual function using anterior segment optical coherence tomography in TGFBI corneal dystrophy
Source: Sci Rep. 2022 Aug 12;12:13759. doi: 10.1038/s41598-022-17738-3 (PMC9374664; doi:10.1038/s41598-022-17738-3)
Supplement: Supplementary file 1 — Supplementary Information 1. [file 41598_2022_17738_MOESM1_ESM.docx]

**Supplement Figure legends**

**Supplement Figure 1**. Fourier components for comparison between the phenotypes and controls within 6-mm. * indicates statistically significant difference.

GCD = granular corneal dystrophy, LCD = lattice corneal dystrophy

**Supplement Figure 2**. Fourier components for comparison between the subtypes and controls within 6-mm. * indicates statistically significant difference.

GCD2 = granular corneal dystrophy type 2, LCD1 = lattice corneal dystrophy type 1, LCD3A = lattice corneal dystrophy type ⅢA, LCD4 = lattice corneal dystrophy type
