# Supplementary figures and images for: Corneal irregularity and visual function using anterior segment optical coherence tomography in TGFBI corneal dystrophy
Source: Sci Rep. 2022 Aug 12;12:13759. doi: 10.1038/s41598-022-17738-3 (PMC9374664; doi:10.1038/s41598-022-17738-3)

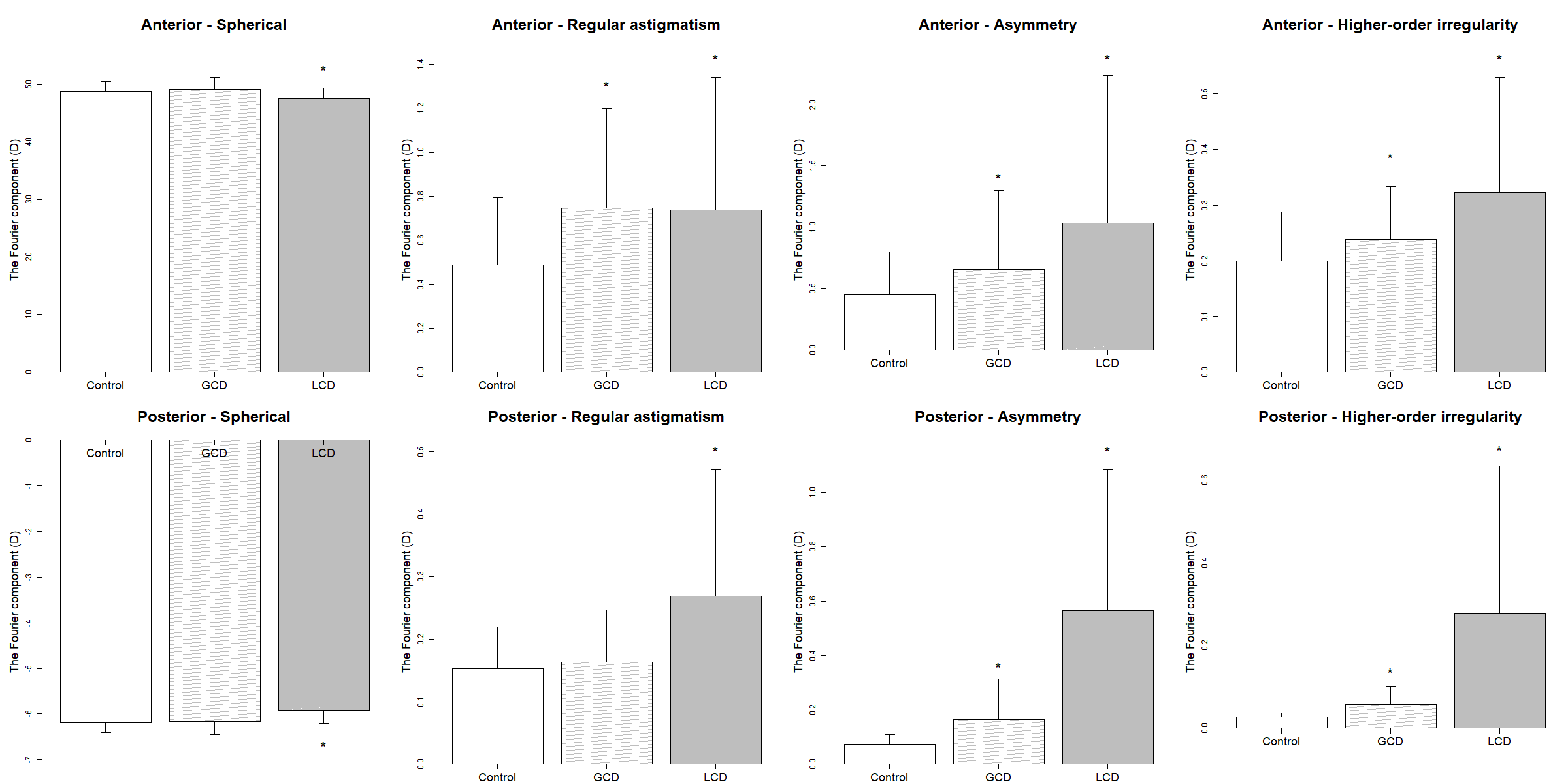

Supplement: Supplementary file 2 — Supplementary Information 2. [file 41598_2022_17738_MOESM2_ESM.tiff]

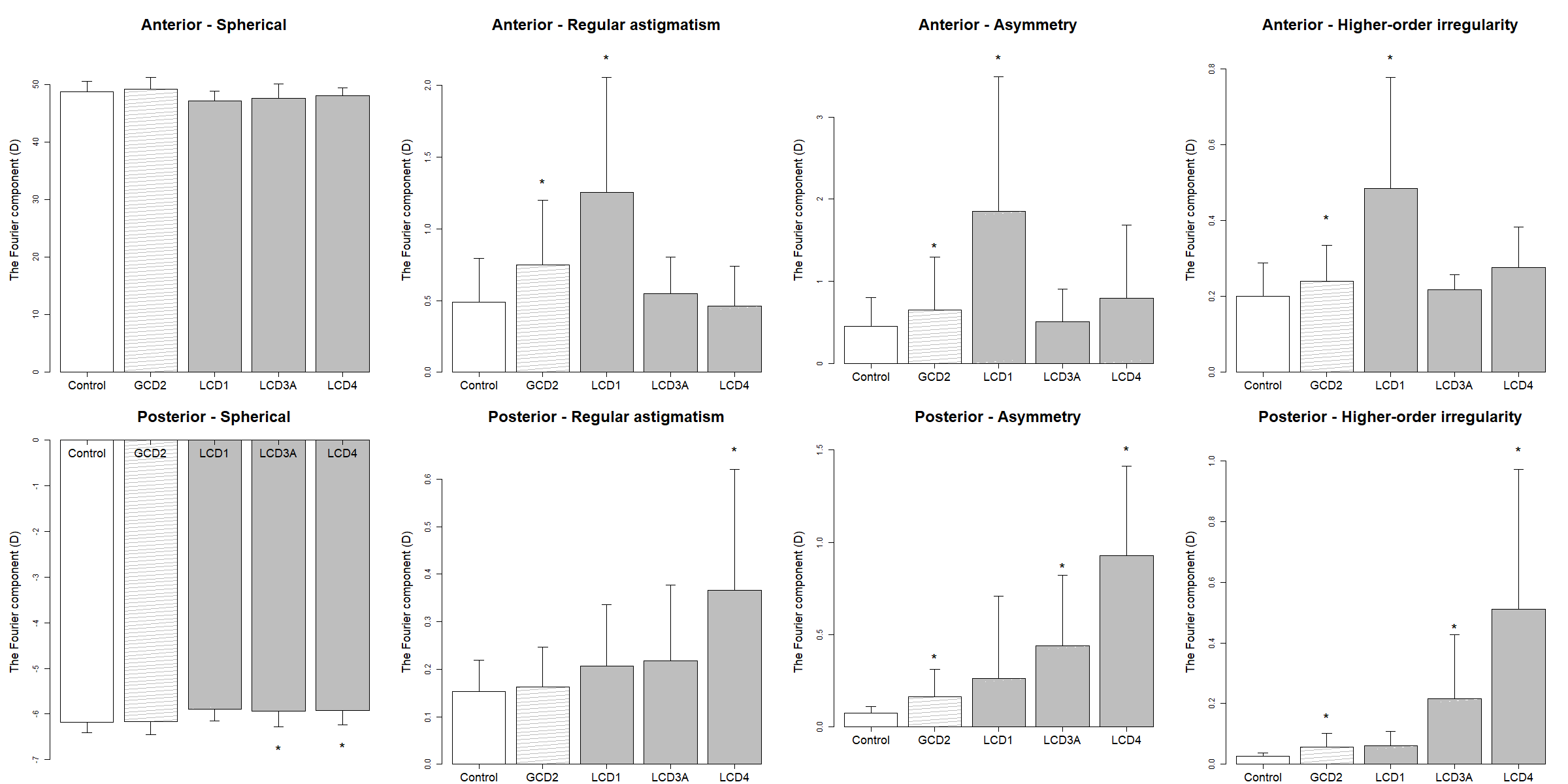

Supplement: Supplementary file 3 — Supplementary Information 3. [file 41598_2022_17738_MOESM3_ESM.tiff]
