# Supplementary material for: Corneal irregularity and visual function using anterior segment optical coherence tomography in TGFBI corneal dystrophy
Source: Sci Rep. 2022 Aug 12;12:13759. doi: 10.1038/s41598-022-17738-3 (PMC9374664; doi:10.1038/s41598-022-17738-3)
Supplement: Supplementary file 4 — Supplementary Information 4. [file 41598_2022_17738_MOESM4_ESM.docx]

**Supplement Table 1**. Each Fourier component within 6-mm

*p* values were calculated using the linear mixed effect model. *, p value < 0.05

|  |  | Anterior | | | |  | Posterior | | | |
| --- | --- | --- | --- | --- | --- | --- | --- | --- | --- | --- |
|  |  | Spherical | Regular | Asymmetry | Higher-order irregularity |  | Spherical | Regular | Asymmetry | Higher-order irregularity |
| Normal | Mean ± SD (D) | 49.8 ± 1.78 | 0.49 ± 0.30 | 0.45 ± 0.35 | 0.20 ± 0.09 |  | -6.17 ± 0.24 | 0.15 ± 0.07 | 0.07 ± 0.04 | 0.03 ± 0.01 |
|  | Range (D) | 44.7, 53.7 | 0.11, 1.59 | 0.11, 2.53 | 0.09, 0.59 |  | -6.82, -5.75 | 0.02, 0.32 | 0.01, 0.17 | 0.01, 0.06 |
| GCD2 | Mean + SD (D) | 49.2 ± 2.12 | 0.75 ± 0.45 | 0.65 ± 0.64 | 0.24 ± 0.09 |  | -6.17 ± 0.30 | 0.16 ± 0.08 | 0.16 ± 0.15 | 0.06 ± 0.04 |
|  | Range (D) | 44.4, 53.3 | 0.17, 2.64 | 0.14, 4.86 | 0.14, 0.79 |  | -6.73, -5.39 | 0.04, 0.44 | 0.02, 0.74 | 0.02, 0.31 |
|  | P value | 0.22 | 0.0025* | 0.022* | 0.014* |  | 0.97 | 0.97 | <0.001* | <0.001* |
| LCD  (total) | Mean ± SD (D) | 47.6 ± 1.81 | 0.74± 0.60 | 1.04 ± 1.2 | 0.32 ± 0.21 |  | -5.92 ± 0.30 | 0.27 ± 0.20 | 0.56 ± 0.52 | 0.28 ± 0.36 |
|  | Range (D) | 42.7, 50.8 | 0.15, 2.91 | 0.10, 4.81 | 0.14, 1.02 |  | -6.79, -5.30 | 0.05, 0.80 | 0.04, 1.97 | 0.02, 1.50 |
|  | P value | 0.026* | 0.017* | <0.001* | <0.001* |  | <0.001* | <0.001* | <0.001* | <0.001* |
| LCD1 | Mean ± SD (D) | 47.1 ± 1.66 | 1.25 ± 0.80 | 1.85 ± 1.64 | 0.48 ± 0.29 |  | -5.89 ± 0.26 | 0.21 ± 0.13 | 0.26 ± 0.45 | 0.06 ± 0.05 |
|  | Range (D) | 44.1, 49.1 | 0.35, 2.91 | 0.15, 4.81 | 0.18, 1.02 |  | -6.20, -5.41 | 0.07, 0.54 | 0.04, 1.59 | 0.02, 0.20 |
|  | P value | 0.24 | <0.001* | <0.001* | <0.001* |  | 0.11 | 0.77 | 0.094 | 0.99 |
| LCD3 | Mean ± SD (D) | 47.6 ± 2.42 | 0.55 ± 0.25 | 0.51 ± 0.40 | 0.22 ± 0.04 |  | -5.94 ± 0.34 | 0.22 ± 0.16 | 0.44 ± 0.38 | 0.22 ± 0.21 |
|  | Range (D) | 42.7, 50,4 | 0.19, 1.05 | 0.10, 1.48 | 0.14, 0.28 |  | -6.39, -5.30 | 0.05, 0.54 | 0.05, 1.15 | 0.03, 0.79 |
|  | P value | 0.22 | 0.95 | 0.97 | 0.97 |  | 0.046* | 0.19 | <0.001* | 0.0023* |
| LCD4 | Mean ± SD (D) | 48.1 ± 1.30 | 0.46 ± 0.28 | 0.79 ± 0.89 | 0.28 ± 0.11 |  | -5.92 ± 0.31 | 0.37 ± 0.25 | 0.93 ± 0.49 | 0.51 ± 0.46 |
|  | Range (D) | 46.7, 50.8 | 0.15, 0.86 | 0.13, 3.56 | 0.15, 0.52 |  | -6.79, -5.55 | 0.07, 0.80 | 0.17, 1.97 | 0.07, 1.50 |
|  | P value | 0.49 | 0.99 | 0.22 | 0.22 |  | 0.016* | <0.001* | <0.001* | <0.001* |

SD= standard deviation, GCD2 = granular corneal dystrophy type 2, LCD = lattice corneal dystrophy, LCD1 = lattice corneal dystrophy type 1, LCD3 = lattice corneal dystrophy type ⅢA, LCD4 = lattice corneal dystrophy type Ⅳ

**Supplement Table 2**. Association between BCVA and Fourier components within 6-mm

|  |  | Anterior | | | |  | | Posterior | |  | | Total corneal | | | |
| --- | --- | --- | --- | --- | --- | --- | --- | --- | --- | --- | --- | --- | --- | --- | --- |
|  |  | Spherical | Regular | Asymmetry | Higher-order irregularity |  | Spherical | Regular | Asymmetry | Higher-order irregularity |  | Spherical | Regular | Asymmetry | Higher-order irregularity |
|  |  |  |  |  |  |  |  |  |  |  |  |  |  |  |  |
| GCD2 | Coefficient | -0.013 | 0.023 | 0.060 | -0.024 |  | 0.035 | -0.16 | 0.035 | -0.16 |  | -0.016 | 0.031 | 0.070 | -0.17 |
|  | P value | 0.31 | 0.67 | 0.32 | 0.94 |  | 0.71 | 0.69 | 0.83 | 0.74 |  | 0.28 | 0.61 | 0.27 | 0.57 |
| LCD  (total) | Coefficient | -0.062 | 0.41 | 0.26 | 1.58 |  | 0.32 | 0.62 | -0.0032 | 0.44 |  | -0.060 | 0.51 | 0.26 | 0.71 |
|  | P value | 0.22 | 0.0052* | <0.001* | <0.001* |  | 0.24 | 0.074 | 0.99 | 0.040 |  | 0.28 | <0.001* | <0.001* | <0.001* |
| LCD1 | Coefficient | -0.068 | 0.46 | 0.32 | 1.58 |  | 0.80 | -1.76 | -0.35 | -3.81 |  | -0.064 | 0.50 | 0.33 | 1.48 |
|  | P value | 0.53 | 0.020* | <0.001* | 0.017* |  | 0.45 | 0.33 | 0.47 | 0.38 |  | 0.57 | 0.0081 | <0.001* | 0.032 |
| LCD3 | Coefficient | -0.048 | 0.59 | 0.35 | 3.94 |  | 0.056 | -0.31 | 0.11 | 0.86 |  | -0.054 | 0.39 | 0.26 | 0.97 |
|  | P value | 0.68 | 0.34 | 0.11 | 0.15 |  | 0.94 | 0.72 | 0.80 | 0.12 |  | 0.66 | 0.51 | 0.18 | 0.07 |
| LCD4 | Coefficient | -0.15 | 0.084 | 0.15 | 1.74 |  | 0.28 | 0.93 | 0.28 | 0.51 |  | -0.12 | 0.77 | 0.15 | 0.58 |
|  | P value | 0.28 | 0.85 | 0.20 | 0.092 |  | 0.47 | 0.0070* | 0.35 | 0.028* |  | 0.43 | 0.13 | 0.28 | 0.018 |

Coefficients and *p* values were calculated using the linear mixed effect model. *, p value < 0.05

BCVA = best-corrected visual acuity, GCD2 = granular corneal dystrophy type 2, LCD (total) = lattice corneal dystrophy (Includes all subtypes), LCD1 = lattice corneal dystrophy type 1, LCD3 = lattice corneal dystrophy type ⅢA, LCD4 = lattice corneal dystrophy type Ⅳ
